# JAZ7 negatively regulates dark-induced leaf senescence in *Arabidopsis*

**DOI:** 10.1093/jxb/erv487

**Published:** 2015-11-07

**Authors:** Juan Yu, Yixiang Zhang, Chao Di, Qunlian Zhang, Kang Zhang, Chunchao Wang, Qi You, Hong Yan, Susie Y. Dai, Joshua S Yuan, Wenying Xu, Zhen Su

**Affiliations:** ^1^State Key Laboratory of Plant Physiology and Biochemistry, College of Biological Sciences, China Agricultural University, Beijing 100193, PR China; ^2^Department of Plant Pathology and Microbiology, Texas A&M University, College Station, TX 77843, USA; ^3^Department of Veterinary Pathobiology, Texas A&M University, College Station, TX 77843, USA

**Keywords:** Arabidopsis, AtJAZ7, COI1, MYC2, dark-induced leaf senescence, transcriptomics.

## Abstract

JASMONATE ZIM-domain (JAZ) proteins play important roles in plant defence and growth by regulating jasmonate signalling. Through data mining, we discovered that the *JAZ7* gene was up-regulated in darkness. In the dark, the *jaz7* mutant displayed more severe leaf yellowing, quicker chlorophyll degradation, and higher hydrogen peroxide accumulation compared with wild-type (WT) plants. The mutant phenotype of dark-induced leaf senescence could be rescued in the *JAZ7*-complemented and -overexpression lines. Moreover, the double mutants of *jaz7 myc2* and *jaz7 coi1* exhibited delayed leaf senescence. We further employed GeneChip analysis to study the molecular mechanism. Some key genes down-regulated in the triple mutant *myc2 myc3 myc4* were up-regulated in the *jaz7* mutant under darkness. The Gene Ontology terms ‘leaf senescence’ and ‘cell death’ were significantly enriched in the differentially expressed genes. Combining the genetic and transcriptomic analyses together, we proposed a model whereby darkness can induce JAZ7, which might further block MYC2 to suppress dark-induced leaf senescence. In darkness, the mutation of JAZ7 might partially liberate MYC2/MYC3/MYC4 from suppression, leading the MYC proteins to bind to the G-box/G-box-like motifs in the promoters, resulting in the up-regulation of the downstream genes related to indole-glucosinolate biosynthesis, sulphate metabolism, callose deposition, and JA-mediated signalling pathways. In summary, our genetic and transcriptomic studies established the JAZ7 protein as an important regulator in dark-induced leaf senescence.

## Highlight

Under darkness, *JAZ7* was up-regulated and the mutant showed a severe leaf senescence phenotype. Genetics and transcriptomic analysis revealed *JAZ7* as an important regulator of dark-induced leaf senescence.

## Introduction

Leaf senescence is a programmed cell death process essential for plant growth and survival. Leaf senescence can be induced by many developmental and environmental factors, such as aging, darkness, hormones, drought, high salinity, extreme temperature, and pathogen attacks (Lim *et al.*, 2007). Dark-induced senescence has frequently been used as a model system to study natural senescence and to promote some typical senescence symptoms such as chlorophyll degradation and protein catabolism (Weaver *et al.*, 1998; Guo and Gan, 2005). Previous studies have revealed many regulators of dark-induced leaf senescence, such as WRKY22 (Zhou *et al.*, 2011). Nitric oxide (NO) is a very important molecule regulating dark-induced leaf senescence, where Nitric Oxide Synthase1 reduces the levels of reactive oxygen species to protect against dark-induced senescence (Guo and Crawford, 2005). NO can regulate dark-induced leaf senescence through EIN2 (ETHYLENE INSENSITIVE 2) in Arabidopsis (Niu and Guo, 2012).

High-throughput technology has greatly facilitated the study of aging- and darkness-related leaf senescence genes. Bhalerao *et al.* (2003) reported differentially expressed genes in autumn leaves by sequencing expressed sequence tags of a cDNA library in aspen (*Populus tremula*). A DNA microarray was used to study the leaf transcriptome of naturally senesced aspen leaves (Andersson *et al.*, 2004). A high-resolution time-course profile of gene expression during development of a single leaf over a 3-week period up to senescence was obtained by microarray analysis (Breeze *et al.*, 2011). Lin and Wu (2004) used the Arabidopsis ATH1 Genome Array to examine global gene expression in dark-induced leaf senescence. More than 800 genes were identified as senescence-associated genes, including large transcription factor families such as NAC, WRKY, C2H2-type zinc finger, AP2/EREBP, and MYB proteins (Gepstein *et al.*, 2003). These senescence-associated genes are closely involved in different plant hormone biosynthesis and signal transduction pathways (Lim *et al.*, 2007). Phytohormones, such as cytokinin, ethylene, abscisic acid, methyl jasmonate, salicylic acid, and auxin, normally act as internal factors to cause leaf senescence through a regulatory network (Aharoni and Lieberman, 1979; Gan and Amasino, 1995; Jing *et al.*, 2005; Lim *et al.*, 2007; Dong *et al.*, 2008; Ghanem *et al.*, 2008).

Jasmonic acid (JA) plays an important role in regulating dark-induced leaf senescence. The bioactive JA–isoleucine (JA–Ile) perception is dependent on the inositol phosphate-potentiated COI1–JAZ co-receptor (Sheard *et al.*, 2010). COI1-dependent JA repression of rubisco activase was suggested to be an essential mechanism for JA-induced leaf senescence (Shan *et al.*, 2010). JASMONATE ZIM-domain (JAZ) proteins were discovered as repressors of JA signalling through the SCF^COI1^-dependent 26S proteasome pathway for protein degradation (Chini *et al.*, 2007; Thines *et al.*, 2007). There are 13 *JAZ* genes in Arabidopsis (Thireault *et al.*, 2015). Both JAZ4 and JAZ8 were found to interact physically with WRKY57 for negative regulation of JA-induced leaf senescence (Jiang *et al.*, 2014). WRKY57 functions as a node of convergence for JA- and auxin-mediated signalling in JA-induced leaf senescence. Generally, the JAZ proteins play important roles in plant defence and growth by regulating JA signalling (Chini *et al.*, 2007; Thines *et al.*, 2007; Yan *et al.*, 2007; Chung *et al.*, 2008; Grunewald *et al.*, 2009; Demianski *et al.*, 2011); however, the function of JAZ proteins in serving as an upstream regulator for leaf senescence has yet to be established.

The majority of the 13 JAZ proteins form homodimers and heterodimers, but interestingly, there is no evidence that any of the JAZ proteins interact with JAZ7 (Chini *et al.*, 2009; Chung and Howe, 2009). Some JAZ proteins (possibly JAZ7) interact directly with TOPLESS (TPL) rather than using NINJA (Novel Interactor of JAZ) (Shyu *et al.*, 2012). Meanwhile, JAZ proteins interact with other transcription regulators. A select set of JAZ proteins (i.e. JAZ1, JAZ3, and JAZ9) can bind EIN3 and EIL1, which are involved in ethylene signalling (Zhu *et al.*, 2011). Overexpression of JAZ1 can disturb the bHLH–MYB interaction between PAP1–TT8 and GL1–GL3 (Qi *et al.*, 2011). Some JAZ proteins such as JAZ1, JAZ8, and JAZ11 interact directly with MYB21 and MYB24 (Song *et al.*, 2011). Direct interaction between JAZ1 and HDA6 has recently been confirmed (Zhu *et al.*, 2011). In addition, JAZ proteins recruit TPL and TPL-related proteins through NINJA for transcription repression (Pauwels *et al.*, 2010). Furthermore, JA signalling is reported to be involved in cambium formation, and COI1, MYC2, JAZ7, and especially JAZ10 are cambium regulators (Sehr *et al.*, 2010). Overall, JAZ proteins regulate plant defence, growth, development, and in particular JA signalling, by interacting with both COI1 and various transcriptional factors. Despite the importance of the *JAZ* gene family, the function of JAZ7 is still largely unknown.

In addition to JAZ proteins, MYC2 has emerged as a master regulator of most aspects of the JA signalling pathway in Arabidopsis (Kazan and Manners, 2013). MYC3 and MYC4 are the closest homologues of MYC2, and were recently identified as targets of JAZ repressors and act additively with MYC2 in regulating the JA-dependent transcriptional response (Fernandez-Calvo *et al.*, 2011; Niu *et al.*, 2011). Most JAZ proteins—including JAZ7—interact with MYC2, MYC3, and/or MYC4 (Fernandez-Calvo *et al.*, 2011; Schweizer *et al.*, 2013). Furthermore, the MYC transcription factors MYC2, MYC3, and MYC4 regulate glucosinolate (GS) biosynthesis, insect performance, and feeding behaviour. MYC2 interacts with the G-box and related sequences, and controls genes activated by JA. A previous study showed that MYC2 binds directly to the promoter of several GS biosynthesis genes *in vivo* (Schweizer *et al.*, 2013). Recent studies have indicated that MYC transcription factors (MYC2, MYC3, and MYC4) are short-lived proteins degraded by the proteasome in darkness (including shade conditions) and stabilized by light and JA. MYC2 protein stability is regulated by phosphorylation-coupled proteolysis through the proteosome (Zhai *et al.*, 2013; Chico *et al.*, 2014). In addition, MYCs are required for JA-mediated defences against the necrotrophic pathogen *Botrytis cinerea* and for the shade-triggered increased susceptibility (Chico *et al.*, 2014). In contrast to MYCs, simulated shade conditions stabilize seven of the ten JAZ repressors and reduce their degradation by JA. Despite the progress, it is still unclear how JAZ proteins interact with MYCs to signal leaf senescence or other processes.

In this study, we integrated a systems biology approach with a classic genetics study to identify JAZ7 involvement in dark-induced leaf senescence. The *JAZ7* gene was significantly up-regulated during darkness. Further analysis of mutant phenotypes for the *JAZ* family genes revealed that a knockout mutant of *JAZ7* resulted in severe leaf senescence under dark treatment. Unlike other *JAZ* genes, very limited information is available for the functions of *JAZ7*. Genetic analysis showed that *JAZ7* is very important for dark-induced leaf senescence. The dark-induced leaf senescence phenotype could be rescued in the *JAZ7*-complemented and -overexpression lines. Further genetic analysis showed that JAZ7 interacted with COI1 and/or MYC2 to regulate dark-induced leaf senescence. We further conducted transcriptomic analysis to dissect the molecular and systems mechanisms underlying the JAZ7-mediated dark-induced leaf senescence.

## Materials and methods

### Plant materials and growth conditions

*Arabidopsis thaliana* (Col-0, *jaz7* mutant line, *JAZ7*-complemented line *35S::JAZ7/jaz7*, and *JAZ7*-overexpression line *35S::JAZ7/WT*, as well as other mutant lines) seeds were surface sterilized and sown on half-strength Murashige and Skoog (MS) medium with 0.8% agar in Petri plates. The seeds were stratified for 3 d at 4 °C and then transferred to a conditioning chamber with a circadian cycle of 16h of light (22 °C) and 8h of darkness (19 °C). The Arabidopsis seedlings were transferred to soil 10 d after germination.

### Identification of the *jaz7* T-DNA insertion mutant

The *jaz7* mutant (WiscDsLox7H11) contained a T-DNA insertion in the second exon (see Fig. 2A). Homozygous T-DNA insertion mutant plants were confirmed by PCR using a combination of a T-DNA border primer (LB: 5'-GCGTGGACCGCTTGCTGCAACT-3') and gene-specific primers (LP: 5'-CATCATCAAAAACTGCGACAAGCC-3', and RP: 5'-GGTAACGGTGGTAAGGGGAAGT-3').

### Construction of transgenic Arabidopsis line JAZ7-complemented line *35S::JAZ7/jaz7* and JAZ7-overexpression line *35S::JAZ7/WT*

In order to generate the genetic complemented line *35S::JAZ7/jaz7* and overexpression line *35S::JAZ7/WT*, the ORF for the *JAZ7* gene was isolated by PCR using the forward primer 5'-GCTCTAGAATGATCATCATCATCAAAAACTGC-3' and reverse primer 5'-GGGGTACC CTATCGGTAACGGTGGTAAG-3'. The ORF (447bp) of *JAZ7* was inserted into the super-1300 vector in the *Xba*I and *Kpn*I sites under the control of a constitutive cauliflower mosaic virus 35S promoter. The construct was verified by sequencing and introduced into the *Agrobacterium tumefaciens* GV3101 strain. The Arabidopsis plants were transformed using the floral infiltration method (Clough and Bent, 1998). Transgenic plants were selected by hygromycin resistance and confirmed by PCR. The homozygous T_2_ seeds of transgenic plants were used for further analysis.

### Double-mutant construction

The Arabidopsis double-mutant plants *jaz7 coi1* and *jaz7 myc2* were constructed by the manual cross-pollination method (Li *et al.*, 1994). Those homozygous for the single mutation *jaz7* (WiscDsLox7H11), *coi1* (SALK_045434), and *myc2* (SALK_017005C) were used. After self-pollinate of F_1_ plants, the homozygous double mutants were identified from the F_2_ generation by PCR using a combination of the T-DNA border primer LB and gene-specific primers LP/RP. The homozygous F_3_ seeds were used for further analysis.

### Dark treatment for leaf senescence, 3,3'-diaminobenzidine (DAB) staining, and chlorophyll content measurement

Arabidopsis plants were grown in soil for 4 weeks, and the fifth or sixth rosette leaf was detached for dark treatment. The detached leaves were incubated in Petri dishes wrapped in aluminium foil and containing two layers of filter paper soaked in 15ml of distilled water.

The hydrogen peroxide (H_2_O_2_) staining agent DAB (Sigma-Aldrich) was dissolved in water and adjusted to pH 3.8 with KOH. The staining process was as described in Bowling *et al.* (1997). Quantitative measurement of H_2_O_2_ production was performed using an Amples Red H_2_O_2_/peroxidase assay kit (Molecular Probes) and the whole procedure followed the manufacturer’s instructions. An ANOVA test of pair-wise comparisons between all genotypes under the treatments was performed using the Excel packages (Brady *et al.*, 2015).

Chlorophyll relative content was measured using a SPAD-502 chlorophyll meter. More than 30 leaves were tested to make a distribution analysis. Each treated leaf was measured three to four times on different areas. The SPAD readings were used directly to quantify chlorophyll content (Uddling *et al.*, 2007; Ling *et al.*, 2011; Ishimaru *et al.*, 2012; Omondi and Kniss, 2014).

### Sample treatment and RNA extraction

Three-week-old Arabidopsis plants grown in soil were used for dark treatment. The control plants were kept under regular growth conditions as described previously, with a diurnal cycle of 16h of light (22 °C) and 8h of darkness (19 °C). The plants for dark treatment were transferred to a continuously dark room for 3 d. After treatment, at 10 am (2h after lights on), dark-treated and control plants were harvested and quickly placed in liquid nitrogen for total RNA extraction.

Total RNA was extracted using TRIzol reagent (Invitrogen). About 200mg of plant material was grounded in liquid nitrogen and mixed with 1ml of TRIzol. The mixture was incubated at room temperature for 5min, and then 0.2ml of chloroform was added and the mixture incubated at room temperature for 10min. The resultant mixture was centrifuged at 12 000*g* at 4 °C for 10min. The supernatant was transferred to a 1.5ml Eppendorf tube and an equal volume of isopropyl alcohol was added, followed by centrifugation. The RNA was washed with 75% ethanol and dissolved in 100 μl of RNase-free water. All RNA samples were then purified using an RNeasy Mini Kit (Qiagen) following the manufacturer’s instructions. The concentration of RNA was measured with an Eppendorf Biophotometer based on *A*_260_/*A*_280_ and *A*_260_/*A*_230_ ratios.

### Real-time RT-PCR

For real-time reverse transcription (RT-PCR analysis, 2 µg of total RNA was used to synthesize cDNA with an M-MLV Reverse Transcription Reagents Kit (Invitrogen). The cDNA samples were diluted to 2ng μl^–1^ for real-time RT-PCR. Triplicate quantitative assays were performed on 8ng of cDNA using the SYBR Green Master mix with an ABI 7900 sequence detection system according to the manufacturer’s protocol (Applied Biosystems). Primers were designed by Primer3 (http://frodo.wi.mit.edu/) and are listed in Supplementary Table S5 at *JXB* online. The relative quantitation method (ΔΔ*C*_T_) was used to evaluate quantitative variation among replicates. Two reference genes, 18S rRNA and *actin7* (At5g09810), were applied as internal controls to normalize all data for the real-time RT-PCR experiments. The primers of the two reference genes are listed in Supplementary Table S5.

### Affymetrix GeneChip experiment

For Affymetrix GeneChip analysis, 8 μg of total RNA for each sample was used to prepare biotin-labelled cRNA targets. All the procedures for cDNA and cRNA synthesis, cRNA fragmentation, hybridization, washing and staining, and scanning were conducted according to the GeneChip Standard Protocol (Eukaryotic Target Preparation). In this experiment, a Poly-A RNA Control kit and a One-Cycle cDNA Synthesis kit were applied. The Affymetrix ATH1 GeneChip was used for this experiment. Affymetrix GCOS software was used for data normalization and comparative analysis. The overall intensities of all probe sets of each array were scaled to 500 to guarantee that hybridization intensity of all arrays was equivalent. A one-way ANOVA test was applied to identify the differentially expressed probe sets through Partek Genomics Suite 6.3. The annotation of each probe set in the Affymetrix ATH1 GeneChip was provided by BLAST from Arabidopsis TAIR10 (http://www.arabidopsis.org)

### Promoter and gene function analysis

The 2kb promoter sequence of each gene was obtained from TAIR (http://www.arabidopsis.org). The Arabidopsis Promoter Element Discovery Tools (http://stan.cropsci.uiuc.edu/tools.php) and PLACE database (http://www.dna.affrc.go.jp/PLACE/) were used for common motif searching.

Gene ontology (GO) enrichment analysis was performed using the agriGO website (Du *et al.*, 2010) and gene set enrichment analysis using plantGSEA (Yi *et al.*, 2013).

## Results and discussion

### Darkness induces JAZ gene expression

**Fig. 1.**
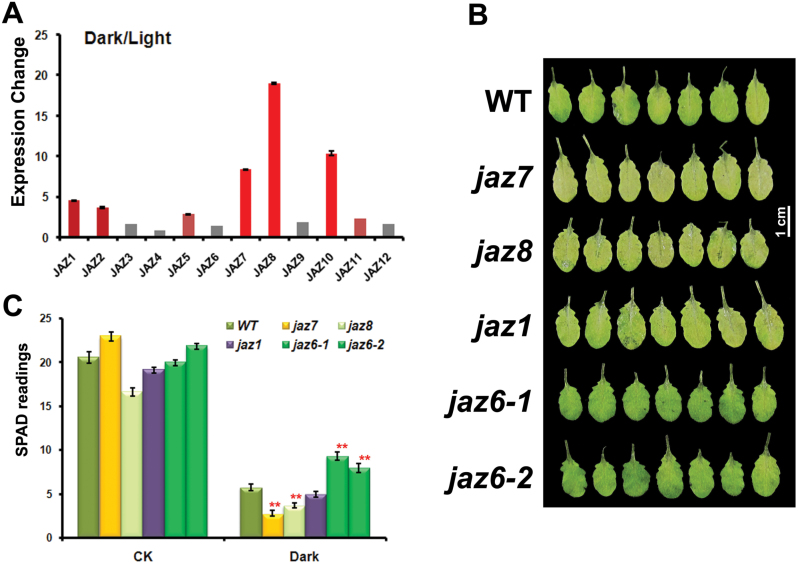
Characterization of the response of *JAZ* family genes to dark-induced leaf senescence. (A) Real-time RT-PCR detection of the expression changes of *JAZ* genes under dark treatment. Total RNA was obtained from leaves of 4-week-old WT plants grown under dark or normal conditions for 3 d. The results show the fold expression change in the dark relative to growth in the light. (B) Rosette leaves of several *JAZ* mutants under dark treatment. The fifth and sixth leaves were detached from the different mutant plants, incubated with wetted filter paper in dark conditions for 118h, and photographed. (C) The chlorophyll content (SPAD readings) of leaves from JAZ mutants under dark treatment and control conditions for 118h. *jaz7*, WiscDsLox7H11; *jaz8*, WiscDsLox255G12; *jaz1*, SALK_011957; *jaz6-1*, SALK_136462; *jaz6-2*, SALK_038013. SPAD readings were determined using a SPAD-502 chlorophyll meter. **P<0.01 (significant difference between mutants and WT according to Student’s *t*-test). The error bars in (A) and (C) represent the standard error of replicates.

We first discovered the potential role of JAZ proteins in dark-induced leaf senescence through data mining of previously published transcriptomics data for dark-induced leaf senescence (Lin and Wu, 2004). *JAZ* genes were found to be significantly induced under dark treatment, and nine out of 12 *JAZ* genes showed more than 2-fold up-regulation for either 1 or 5 d of dark treatments (Supplementary Table S1 at *JXB* online). The expression patterns of Arabidopsis *JAZ* family genes under dark treatment were further investigated using real-time RT-PCR analysis (Fig. 1A and Supplementary Table S1). Seven *JAZ* genes were significantly up-regulated by dark treatment. In addition, Parlitz *et al.* (2011) showed that *JAZ7* and *JAZ10* were significantly induced after 2, 4, and 6 d of dark treatment (Supplementary Table S1). The real-time RT-PCR data and data mining of transcriptomics results both revealed that *JAZ* genes were induced in the dark and might be involved in dark-induced leaf senescence.

### Genetic analysis reveals JAZ7 as an important regulator of dark-induced leaf senescence

Genetic analysis of dark-induced leaf senescence phenotypes for *jaz1*, *jaz6*, *jaz7*, and *jaz8* was further carried out using T-DNA insertion mutant lines (Fig. 1B, C). After 5 d of dark treatment, the detached leaves of *jaz7* mutant lines showed a significantly more severe leaf senescence phenotype, with yellow leaves and less chlorophyll content compared with WT (Col-0). Mutant lines of *jaz1* and *jaz8* also showed less chlorophyll content than WT, while mutants of the two *jaz6* alleles showed resistance to senescence. Among all mutant lines, the *jaz7* mutant showed the most statistically significant decrease in chlorophyll content compared with WT. This result highlighted the important role of JAZ7 as a potential negative regulator in dark-induced leaf senescence.

**Fig. 2.**
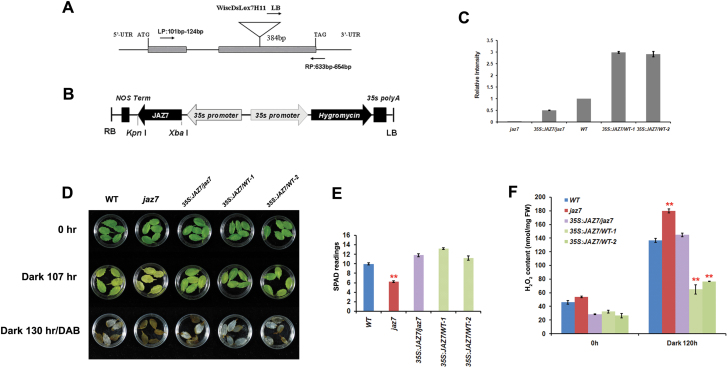
Phenotype analysis of *jaz7* mutant, *JAZ7*-complemented (*35S::JAZ7/jaz7*), WT, and *JAZ7*-overexpression (*35S::JAZ7/WT*) plants under dark treatment. (A) T-DNA insertion position of the *jaz7* mutant line at 384bp from the 5'-untranslated region (UTR). Boxes and lines represent exons and introns, respectively. (B) Constructs used for transformation of the *35S::JAZ7/jaz7*-complemented line and *JAZ7*-overexpression lines. (C) Real-time RT-PCR analysis of *JAZ7* gene expression in the *jaz7* mutant line, WT, complemented line *35S::JAZ7/jaz7*, and overexpression line *35S::JAZ7/WT*. (D) Fifth or sixth detached rosette leaves from the *jaz7* mutant, *35S::JAZ7/jaz7*, WT, and *35S::JAZ7/WT* lines under dark treatment for 4–5 d in water, and DAB staining of the treated leaves. (E) Chlorophyll content (SPAD readings) of leaves after dark treatment for 107h. Error bars represent the standard error of >50 leaves at the same stage. (F) H_2_O_2_ content of leaves from the *jaz7* mutant, *35S::JAZ7/jaz7*, WT, and *35S::JAZ7/WT* lines before and after dark treatment. The SPAD reading was determined using a SPAD-502 chlorophyll meter. FW, fresh weight of leaf tissues. **P<0.01 (significant difference between mutants and WT according to Student’s *t*-test). The error bars in (C) and (F) represent the standard error of replicates.

Genetic complementation of the *jaz7* mutant phenotype was carried out with two types of transgenic strategies: *35S::JAZ7/jaz7* to complement the *JAZ7* mutation in the *jaz7* mutant, and *35S::JAZ7/WT* to overexpress the *JAZ7* gene in WT. Both types of transformants were validated by real-time RT-PCR (Fig. 2A–C). The *35S::JAZ7/jaz7* successfully rescued leaf senescence by showing a similar phenotype to WT (Fig. 2D, 2E). In addition, *35S::JAZ7/WT* displayed a significantly delayed leaf senescence in darkness, further confirming the role of JAZ7 as a negative regulator of dark-induced leaf senescence (Fig. 2D). Among all genetic lines, the *jaz7* mutant had the lowest chlorophyll level in leaves, and overexpression lines had the highest (Fig. 2E). Overall, the genetic complementation experiments and overexpression studies verified the role of JAZ7 in negative regulation of dark-induced leaf senescence.

### H_2_O_2_ is involved in JAZ7-mediated dark-induced leaf senescence

The signalling pathway of JAZ7-mediated dark-induced leaf senescence was further studied from several aspects. First, the role of H_2_O_2_ in JAZ7 signalling for dark-induced leaf senescence was studied. H_2_O_2_ has been established to be important in signalling during stress responses and can be involved in senescence (Orozco-Cardenas *et al.*, 2001; Ali *et al.*, 2007; Aftab *et al.*, 2011). DAB staining was used to detect H_2_O_2_ accumulation under dark treatment, and most *jaz7* mutant leaves stained dark brown, while WT and *35S::JAZ7/jaz7* leaves were lighter in colour, and the *35S::JAZ7/WT* lines showed the least staining (Fig. 2D). The quantitative assay suggested that the *jaz7* mutant produced more H_2_O_2_ than WT and *35S::JAZ7/jaz7*. The *JAZ7* overexpression lines had the lowest H_2_O_2_ levels after 120h of dark treatment (Fig. 2F). Using a pair-wise ANOVA test, the genotypic variation (mutation and overexpression of *JAZ7* gene) was found to significantly alter the H_2_O_2_ level under darkness (Supplementary Table S2 at *JXB* online). The H_2_O_2_ measurement results indicated that the JAZ7 protein might suppress leaf senescence by reducing H_2_O_2_ levels.

### COI1 genetically interacts with JAZ7 to regulate dark-induced leaf senescence

**Fig. 3.**
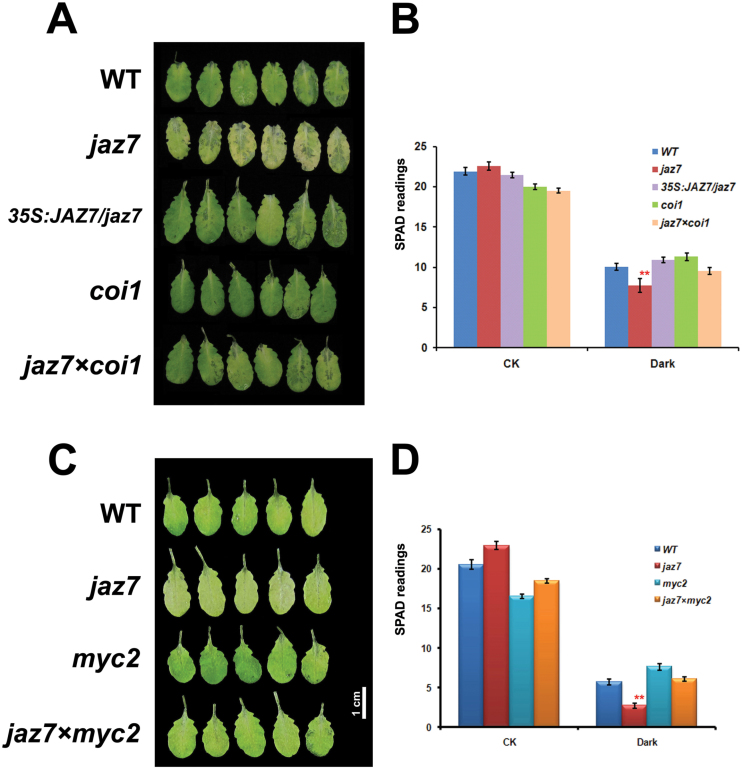
Phenotype analysis of the *jaz7* and double-mutant (*jaz7 coi1* and *jaz7 myc2*) plants under dark treatment. (A) Leaves of WT, *jaz7* mutant, *35S::JAZ7/jaz7* mutant, *coi1* mutant, and *jaz7 coi1* double-mutant lines under dark treatment. The rosette leaves were detached from different mutant plants and photographed after incubation in darkness for 118h. (B) Chlorophyll content (SPAD readings) of the leaves of *jaz7* and *coi1* mutants after dark treatment for 118h. (C) Leaves of WT, *jaz7* mutant, *myc2* mutant, and *jaz7 myc2* double mutants after dark treatment for 118h. (D) Chlorophyll content (SPAD readings) of leaves of *jaz7* and *myc2* mutants after dark treatment for 118h. **P<0.01 (significant difference between mutants and WT according to Student’s *t*-test). The error bars in (B) and (D) represent the standard error of replicates.

Considering that JAZ proteins are repressors of JA signalling through the SCF^COI1^-dependent 26S proteasome pathway (Chini *et al.*, 2007; Thines *et al.*, 2007), we further studied how *JAZ7* interacted genetically with *COI1* during dark-induced leaf senescence. The *jaz7 coi1* double mutant was generated to investigate whether the JAZ7-mediated dark-induced leaf senescence was COI1 dependent or not (Supplementary Fig. S1A, B at *JXB* online). The leaf senescence phenotype for the *jaz7* mutant was partially rescued by mutation of COI1 (Fig. 3A). Basically, after 5 d of darkness treatment, the *jaz7 coi1* double mutant had a similar phenotype to WT plants under darkness, while the *coi1* mutant plants remained green. The chlorophyll level was significantly reduced after dark treatment in all lines (Fig. 3B). The rate of chlorophyll degradation in the *jaz7* mutant was much faster than in the *jaz7 coi1* double-mutant, WT, overexpression line, and *coi1* mutant plants. The results suggested that in the *jaz7 coi1* double mutant, JAZ7 is knocked down but that the other JAZ proteins might be stabilized by the *coi1* mutant, thus repressing MYCs and overcoming the lack of JAZ7.

### MYC2 might be a downstream target of JAZ7

We further studied the downstream target for JAZ7 and explored the genetic interaction of *JAZ7* with *MYC* genes. MYC2 serves as a transcriptional activator of JA responses and JAZ7 physically interacts with MYC2, MYC3, and MYC4 (Fernandez-Calvo *et al.*, 2011; Schweizer *et al.*, 2013). Darkness/shade conditions can stabilize JAZ7 and destabilize MYC proteins (Chico *et al.*, 2014). The rosette leaves detached from WT, *jaz7* mutant, *myc2* mutant, and *jaz7 myc2* double-mutant plants, were treated in darkness to evaluate dark-induced leaf senescence (Fig. 3C, D and Supplementary Fig. S1C, D). The *jaz7* mutant displayed a strong yellowing phenotype at 5 d, while the *myc2* mutant and *jaz7×myc2* double mutant showed comparatively delayed leaf senescence and chlorophyll degradation as compared with *jaz7* mutant. The result indicated that the mutation of *MYC2* could partially rescue the dark-induced chlorophyll degradation in the *jaz7* mutant, and further suggested that MYC2 might be the target of JAZ7 in the signalling of dark-induced leaf senescence.

### Transcriptome analysis of *jaz7* mutant and WT plants under darkness

**Fig. 4.**
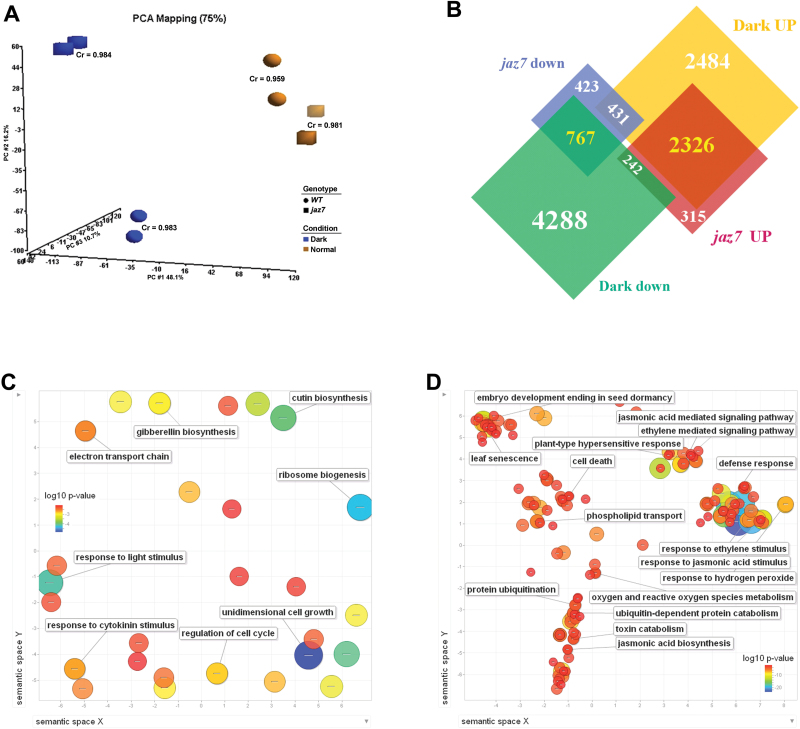
Summary of the differential expression probe sets between WT and *jaz7* under normal and dark treatments. (A) PCA for the samples based on the raw data of the expression profiling. (B) Venn diagrams illustrate the differential expression probe sets under normal and dark treatments in WT and *jaz7* plants. (C) GO enrichment analysis of the 767 probe sets up-regulated in WT and light conditions. (D) GO enrichment analysis of the 2326 probe sets up-regulated in *jaz7* and dark conditions.

Transcriptomics was used to further explore the downstream network regulated by JAZ7 for dark-induced leaf senescence. We used the Arabidopsis ATH1 GeneChip to investigate the genome-wide gene expression differences in dark-induced senescence between WT and *jaz7*. Rosette leaves of Arabidopsis WT and *jaz7*, after 3 d of dark treatment and control conditions, were harvested for GeneChip hybridization, with two independent biological replicates for each sample (Supplementary Table S3 at *JXB* online). Principal component analysis (PCA) showed that the replicate chips of each sample were similar to each other, and the correlation coefficients of the expression profiling for replicate chips were >0.95 (Fig. 4A). The PCA results also showed that the difference between WT and *jaz7* was much higher in darkness than in control conditions (Fig. 4A). Next, we applied ANOVA to identify the differentially expressed probe sets with the cut-off values of ≥1.6 in fold change and of *P*≤0.05. We first checked the differential expression pattern of *DIN* (dark-inducible) genes (Fujiki *et al.*, 2001). The eight *DIN* genes were all significantly induced under dark treatment both in WT and *jaz7*; *DIN9* and *DIN11* were up-regulated in *jaz7* samples compared with WT in darkness (Supplementary Table S4 at *JXB* online). We divided the differentially expressed genes into two categories: dark- and genotype-influenced genes (Fig. 4B). Genes differentially expressed between genotypes fell into two categories: 2883 up-regulated and 1621 down-regulated genes in *jaz7* samples compared with WT. The dark-influenced genes were also divided into two categories: 5241 up-regulated and 5297 down-regulated genes (Fig. 4B). The Venn diagram shows the intersection of the four categories: of the 1621 down-regulated genes in *jaz7*, 767 (>45%) were down-regulated and 431 (about 25%) were up-regulated after dark treatment, while of the 2883 up-regulated genes in *jaz7*, 2326 (>80%) were up-regulated and 242 (<10%) were down-regulated by dark treatment (Fig. 4B). We proposed that two groups of genes were the key factors causing the difference between WT and the *jaz7* mutant: 767 genes that were down-regulated by darkness and had lower expression in the *jaz7* mutant (defined as group I), and 2326 genes that were highly expressed in the *jaz7* mutant and up-regulated by darkness (group II). Therefore, further GO enrichment analyses based on these two groups were carried out. The result for group I (767 genes, Fig. 4C and Supplementary Table S3) showed such enriched GO terms as ‘response to light stimulus’ [GO: 0009416, false discovery rate (FDR) *P* value=7.77E–5], ‘electron transport chain’ (GO:0022900, FDR *P* value=2.59E–3), ‘ribosome biogenesis’ (GO: 0042254, FDR *P* value=5.28E–5), and ‘cutin biosynthetic process’ (GO:0010143, FDR *P* value=1.05E–4). The enriched GO terms for group II (2326 genes, Fig. 4D and Supplementary Table S3) are also shown and included ‘leaf senescence’ (GO:0010150, FDR *P* value=2.48E–7), ‘response to hydrogen peroxide’ (GO: 0042542, FDR *P* value=2.28E–8), ‘cell death’ (GO: 0008219, FDR *P* value=2.42E–3), ‘defence response’ (GO:0006952, FDR *P* value=1.47E–6), and ‘protein ubiquitination’ (GO:0016567, FDR *P* value=1.15E–4).

Based on the enriched GO terms and important superfamilies in differentially expressed genes between the *jaz7* mutant and WT following dark treatment, we selected some genes that were possibly related to senescence/aging to confirm our GeneChip result, including *JAZ* family genes, cytochrome P450s, and UDP-glucoronosyl/UDP-glucosyl transferase family genes. For the real-time RT-PCR validation, additional biological samples were collected under the same conditions as for the GeneChip analysis. Both 18S rRNA and the housekeeping gene *actin7* (At5g09810) were used as reference genes to calculate the relative expression levels. The result of real-time RT-PCR for the majority of the tested genes confirmed the microarray results (Supplementary Fig. S2 at *JXB* online). The fold change of genes analysed by real-time RT-PCR were not exactly the same as those obtained in the GeneChip data, but the change trends were similar.

### Relevance of JAZ- and MYC-regulated pathways

To elucidate the relevance of the regulations by JAZ7 and MYC transcription factors, the previously published down-regulated transcripts in the triple mutant *myc2 myc3 myc4* (Schweizer *et al.*, 2013) were compared with differentially expressed genes in the *jaz7* mutant versus WT. The 28 genes down-regulated in the triple mutant were significantly up-regulated in the *jaz7* mutant in darkness but not under light conditions (Table 1). These genes included those involved in indole-GS, sulphur metabolism, and JA-related pathways. About half of the genes were relevant to indole-GS and sulphur metabolism, including *ASA1*, *ASB1*, *CYP79B2*, *UGT74B1*, *GGP1*, *SOT16/17*, *APR1*, *APK2*, and *APS1*. These genes are essential for glutathione biosynthesis, which is important for Arabidopsis defence, hypersensitive reactions, and programmed cell death. Schweizer *et al.* (2013) reported that MYC2/MYC3/MYC4 are necessary for direct transcriptional activation of GS biosynthesis genes and play crucial roles in the regulation of defence secondary metabolite production. In the triple mutant *myc2 myc3 myc4*, both aliphatic- and indole-GS biosynthesis genes were significantly down-regulated, as well as those related to the sulphate assimilation pathway. Our ATH1 GeneChip data indicated that the mutation of *JAZ7* elevated only the expression levels of genes related to indole-GS biosynthesis and sulphate metabolism under dark treatment (Table 1). This may be because JA can trigger indole-GS accumulation in Arabidopsis, and the induction of tryptophan and indole-GS transcripts are mainly JA dependent (Brader *et al.*, 2001). Thus, JAZ7 might bind to MYC2/MYC3/MYC4 to prevent the activation of indole-GS biosynthesis and sulphur metabolism genes during dark-induced leaf senescence. Furthermore, the genes related to JA pathways were also enriched (Table 1), including *OPR3*, *ST2A*, *NATA1*, and *MYB21*, as well as oxidation–reduction pathway genes such as 2-oxoglutarate-dependent dioxygenase, which suggested a potential role of JA signalling and interference of JAZ7 with its regulation of MYC-dependent cell death and senescence. Further motif analysis revealed that MYC2-binding motifs [G-box (CACGTG) and G-box-like motif (CACATG)] were over-represented in the 2kb promoter regions of these overlapping genes. The predicted MYC2-binding motif and the MYC2-binding information for these GS genes are given in Table 1.

**Table 1.** MYC2-, MYC3-, and MYC4-regulated genes affected by mutation of *JAZ7* in darkness

Locus ID	MYCs motif (CACNTG)	Col-0/*myc234* ^*a*^		WT dark/*jaz7* dark		WT light/*jaz7* light		Gene name	Gene included in functional category of:				
		Fold change	*P* value	Fold change	*P* value	Fold change	*P* value		Indole derivative metabolism	Glucosinolate metabolism	Sulphur metabolism	Response to JA stimulus	Oxidation– reduction
At5g05730	**✓**	**2.27**	**1.47E–04**	**–4.28**	**7.75E–06**	–1.05	9.22E–01	ASA1	**✓**				
At1g25220	**✓**	**2.25**	**2.95E–02**	**–1.83**	**1.17E–02**	–1.33	5.93E–01	ASB1	**✓**	**✓**			
At4g27070	**✓**	**2.03**	**7.95E–03**	**–2.37**	**1.39E–02**	1.05	9.50E–01	TSB2	**✓**				
At4g39950^*b*^	**✓**	**11.42**	**6.17E–06**	**–4.06**	**5.95E–04**	–1.08	9.52E–01	CYP79B2	**✓**	**✓**	**✓**		
At1g24100^*b*^	**✓**	**3.93**	**5.27E–06**	**–2.21**	**9.39E–03**	1.05	8.49E–01	UGT74B1	**✓**	**✓**	**✓**		
At4g30530	**✓**	**3.22**	**1.90E–05**	**–1.81**	**2.43E–02**	–1.38	3.09E–01	GGP1	**✓**	**✓**	**✓**		
At1g74100^*b*^	**✓**	**4.12**	**4.52E–06**	**–2.28**	**2.64E–02**	–1.76	5.14E–01	SOT16	**✓**	**✓**	**✓**	**✓**	
At1g18590	**✓**	**15.50**	**1.18E–07**	**–1.97**	**2.52E–02**	**–2.06**	**1.80E–02**	SOT17	**✓**	**✓**	**✓**		
At4g39940^*b*^	**✓**	**35.27**	**5.54E–08**	**–2.17**	**5.19E–03**	–1.33	1.04E–01	APK2,AKN2	**✓**	**✓**	**✓**		
At4g04610^*b*^	**✓**	**4.21**	**1.18E–04**	**–8.31**	**4.35E–05**	–1.37	4.46E–01	APR1, PAPS reductase	**✓**	**✓**	**✓**		**✓**
At3g22890	**✓**	**1.99**	**1.14E–03**	**–2.32**	**6.40E–03**	1.26	3.45E–01	APS1	**✓**	**✓**	**✓**		
At1g51760	**✓**	**1.85**	**6.39E–04**	**–2.22**	**6.61E–03**	–2.66	6.22E–02	IAR3, JR3	**✓**			**✓**	
At5g01500		**2.93**	**3.21E–05**	**–3.61**	**1.52E–02**	1.61	5.60E–01	TAAC/PAPST1		**✓**	**✓**		
At4g29700	**✓**	**3.59**	**5.24E–05**	**–2.06**	**2.05E–02**	–1.38	1.98E–01	Alkaline- phosphatase-like protein		**✓**	**✓**		
At5g07010	**✓**	**2.34**	**2.62E–04**	**–2.98**	**1.18E–04**	1.27	9.44E–01	ST2A			**✓**	**✓**	
At2g06050	**✓**	**3.36**	**1.55E–05**	**–2.61**	**2.51E–02**	–1.19	5.47E–01	OPR3				**✓**	**✓**
At2g39030	**✓**	**7.74**	**3.10E–05**	**–2.96**	**1.32E–03**	–1.46	3.73E–01	NATA1				**✓**	
At3g27810	**✓**	**1.89**	**2.43E–03**	**–3.19**	**2.92E–03**	–3.36	4.21E–01	ATMYB21, ATMYB3, MYB21				**✓**	
At1g61120	**✓**	**5.22**	**2.65E–05**	**–4.81**	**1.99E–04**	–1.55	4.58E–01	TPS04				**✓**	
At3g51450	**✓**	**1.91**	**2.00E–03**	**–2.37**	**3.03E–02**	–1.39	2.13E–01	Calcium-dependent phosphotriesterase				**✓**	
At1g06620	**✓**	**2.53**	**5.47E–05**	**–2.31**	**3.75E–03**	–1.35	6.23E–01	2-Oxoglutarate- dependent dioxygenase				**✓**	**✓**
At2g38240	**✓**	**4.47**	**6.37E–05**	**–2.70**	**2.68E–04**	–1.43	8.94E–01	Oxoglutarate/ iron-dependent oxygenase					**✓**
At4g20860	**✓**	**2.29**	**1.74E–03**	**–3.35**	**2.97E–03**	–1.77	8.03E–01	FAD-binding Berberine protein					
At1g74010		**2.39**	**9.69E–03**	**–2.17**	**1.29E–02**	–1.12	9.35E–01	Calcium-dependent phosphotriesterase					
At4g24350	**✓**	**1.92**	**3.88E–04**	**–2.69**	**2.99E–02**	–1.49	3.01E–02	Phosphorylase					
At3g03240	**✓**	**1.94**	**1.52E–03**	**–1.95**	**4.18E–02**	1.20	8.70E–01	α/β-Hydrolases					
At2g25730	**✓**	**1.86**	**1.27E–02**	**–1.87**	**1.23E–02**	–1.29	6.89E–01	Unknown protein					
At5g40210	**✓**	**2.43**	**4.17E–04**	**–2.48**	**1.85E–03**	1.39	3.06E–02	Nodulin MtN21 /EamA-like transporter					

Bold values represent statistically significant changes (fold change ≥1.8 and *P*≤0.05).

^*a*^ Data from Schweizer *et al.* (2013).

^*b*^ The genes were reported with MYC2 bound in the promoter region by ChIP-seq (Schweizer *et al.*, 2013).

In addition, indole-GS biosynthesis is required for the Flg22-induced callose response and Arabidopsis innate immune response (Clay *et al.*, 2009; Schlaeppi *et al.*, 2010). Transcriptome analysis of WT and *jaz7* mutant plants under dark treatment showed that a large number of callose deposition genes were also up-regulated in the *jaz7* mutant. Most of these callose-response genes were related to the indole-GS pathway, such as *TSA1/TRP3*, *CYP79B2*, *UGT74B1*, *CYP81F2*, and *PEN3*. All of the results suggested that the JAZ7-mediated MYC-regulated indole-GS genes were involved in callose deposition, essential for plant innate immunity response, programmed cell death, and leaf senescence.

### Towards a model for JAZ7-mediated dark-induced leaf senescence

**Fig. 5.**
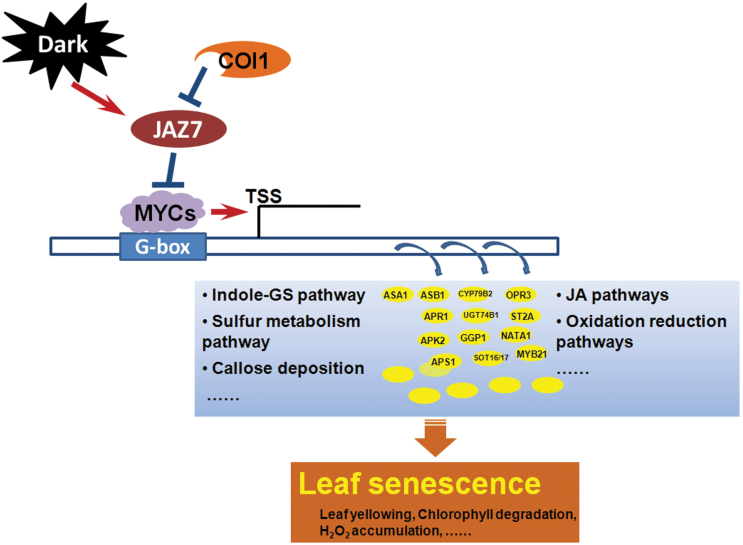
Working model of the role of JAZ7 in dark-induced leaf senescence. Under darkness, expression of the *JAZ7* gene is significantly induced, and the *jaz7* mutant had enhanced dark-induced leaf senescence. JAZ7-mediated dark-induced senescence might be COI1 dependent and antagonistic to regulation by MYCs. During darkness, the mutation of JAZ7 might partially liberate MYCs from suppression, leading the MYCs to bind to the G-box/G-box-like motifs in the promoters and resulting in the up-regulation of the downstream genes related to indole-GS biosynthesis, sulphate metabolism, callose deposition, and JA-mediated signalling pathways.

Together, the genetic and transcriptomic analyses suggested a model (Fig. 5) whereby darkness can induce JAZ7, which might further block MYC2 to suppress dark-induced leaf senescence. The function of JAZ7 may also depend on COI1. In darkness, the mutation of JAZ7 might partially liberate MYC2/MYC3/MYC4 from suppression, leading the MYC proteins to bind to the G-box/G-box-like motifs in the promoters, resulting in up-regulation of the downstream genes related to indole-GS biosynthesis, sulphate metabolism, callose deposition, and JA-mediated signalling pathways. Overall, the genetic and systems biology analyses suggest that JAZ7 plays an essential role in regulating the dark-induced leaf senescence relevant to many physiological processes.

## Supplementary data

Supplementary data are available at *JXB* online.

**Supplementary Fig. S1.** Real-time RT-PCR for target genes in *jaz7*, *coi1*, and *myc2* mutants and double mutants *jaz7 coi1*, *jaz7 myc2*.

**Supplementary Fig. S2.** Real-time RT-PCR for selected probe sets.

**Supplementary Table S1.** Expression pattern of *JAZ* family genes under dark treatment.

**Supplementary Table S2.** ANOVA tables for pair-wise comparisons in Fig. 2F.

**Supplementary Table S3.** GeneChip raw data for WT and *jaz7* samples under normal and dark treatments.

**Supplementary Table S4.** Expression pattern of *DIN* genes for WT and *jaz7* under normal and dark treatments.

**Supplementary Table S5.** List of primers used for real-time RT-PCR.

Supplementary Data
