# Supplementary material for: JAZ7 negatively regulates dark-induced leaf senescence in Arabidopsis
Source: J Exp Bot. 2015 Nov 7;67(3):751–62. doi: 10.1093/jxb/erv487 (PMC4737072; doi:10.1093/jxb/erv487)
Supplement: Supplementary Data [file supp_67_3_751__index.html]

JAZ7 negatively regulates dark-induced leaf senescence in Arabidopsis — JAZ7 negatively regulates dark-induced leaf senescence in Arabidopsis — Supplementary Data 

# JAZ7 negatively regulates dark-induced leaf senescence in *Arabidopsis*

## Supplementary Data

Data files

- supplementary\_Fig.\_S1\_S2\_Tables\_S1\_S2\_\_S4\_S5.pdf - Supplementary Data
- supplementary\_table\_S3.xls - Supplementary Data
