# Supplementary material for: JAZ7 negatively regulates dark-induced leaf senescence in Arabidopsis
Source: J Exp Bot. 2015 Nov 7;67(3):751–62. doi: 10.1093/jxb/erv487 (PMC4737072; doi:10.1093/jxb/erv487)
Supplement: Supplementary Data [file supp_erv487_supplementary_Fig._S1_S2_Tables_S1_S2__S4_S5.pdf]

JAZ7 negatively regulates dark-induced leaf senescence in *Arabidopsis*

SUPPLEMENTARY MATERIAL

Supplementary Figure 1. Real-time RT-PCR for target genes in *jaz7*, *coi1*, *myc2* mutants and double mutant *jaz7×coi1*, *jaz7×myc2*

A. Expression level of *COI1* in *coi1* mutant and double mutant *jaz7×coi1*.
B. Expression level of *JAZ7* in *jaz7* mutant and double mutant *jaz7×coi1*.
C. Expression level of *MYC2* in *myc2* mutant and double mutant *jaz7×myc2*.
D. Expression level of *JAZ7* in *jaz7* mutant and double mutant *jaz7×myc2*.

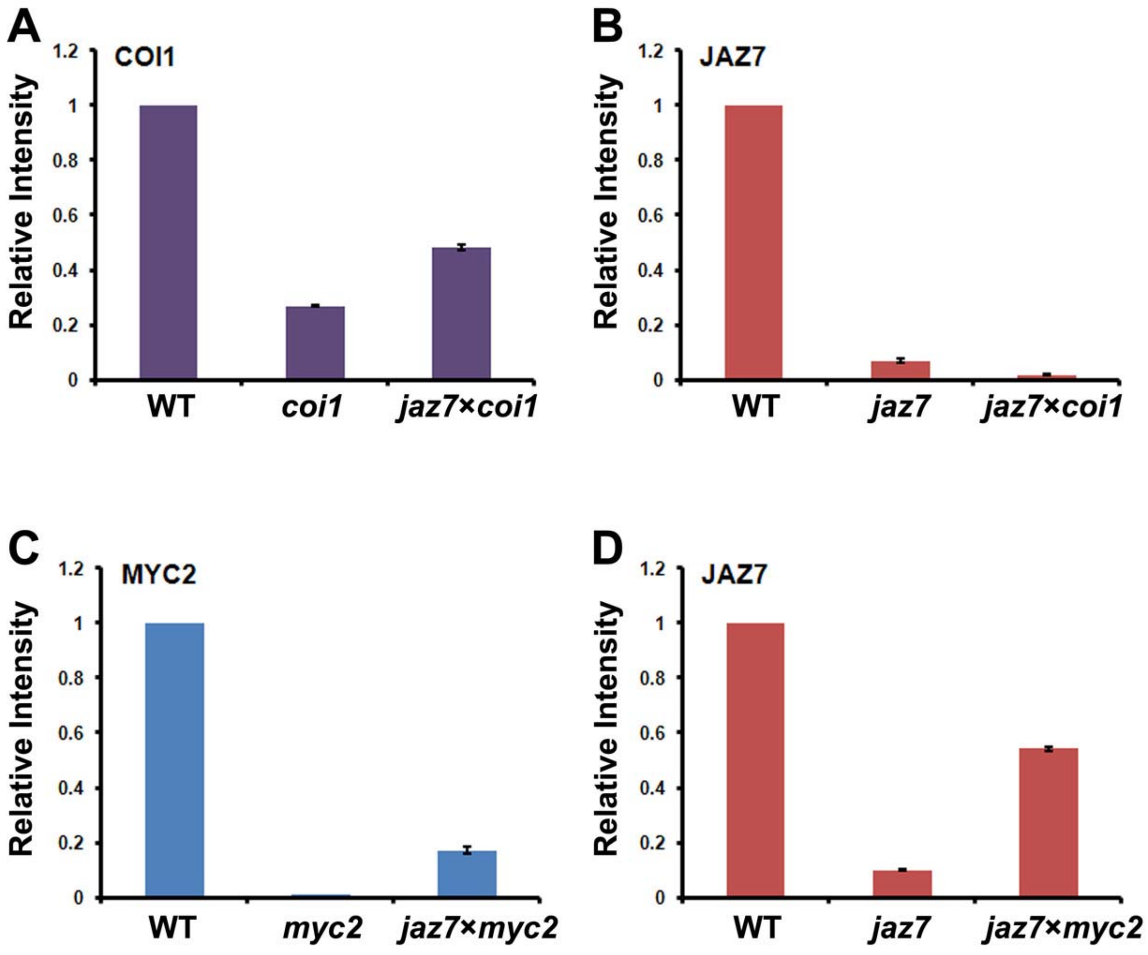

Supplementary Figure 2. Real-time RT-PCR for selected probe sets

At5g09810--Actin7; At2g34600--JAZ7; At1g30135--JAZ8; At1g17380--JAZ5; At1g72450--JAZ6; At1g19180--JAZ1; At1g74950--JAZ2; At3g17860--JAZ3; At1g48500--JAZ4; At1g70700--JAZ9; At5g13220--JAZ10; At3g43440--JAZ11; At5g20900--JAZ12; At2g39730--RCA.2; At2g39730--RCA.1; At3g06490--MYB108; At3g48520--CYP94B3; At3g46660-- UGT76E12; At3g45970--ATEXLA1; At4g33030--SQD1; At4g36810--GGPS1; At4g16780--ATHB-2; At1g22740--RABG3B; At3g17790--PAP17; At1g29395--COR414-TM1; At5g61210--SNAP33; At1g07990--SIT4; At1g29370-- Kinase-related protein of unknown function (DUF1296); At5g47480-- RGPR-related protein; At1g63770-- Peptidase M1 family protein; At2g38020--VCL1; At2g20960--pEARLI4; At3g54540--ATGCN4; At4g27130-- Translation initiation factor SUI1 family protein; At2g39940--COI1; At1g32640--MYC2; At4g17880--MYC4; At5g46760--MYC3

The error bars represent the standard error of three replicates.

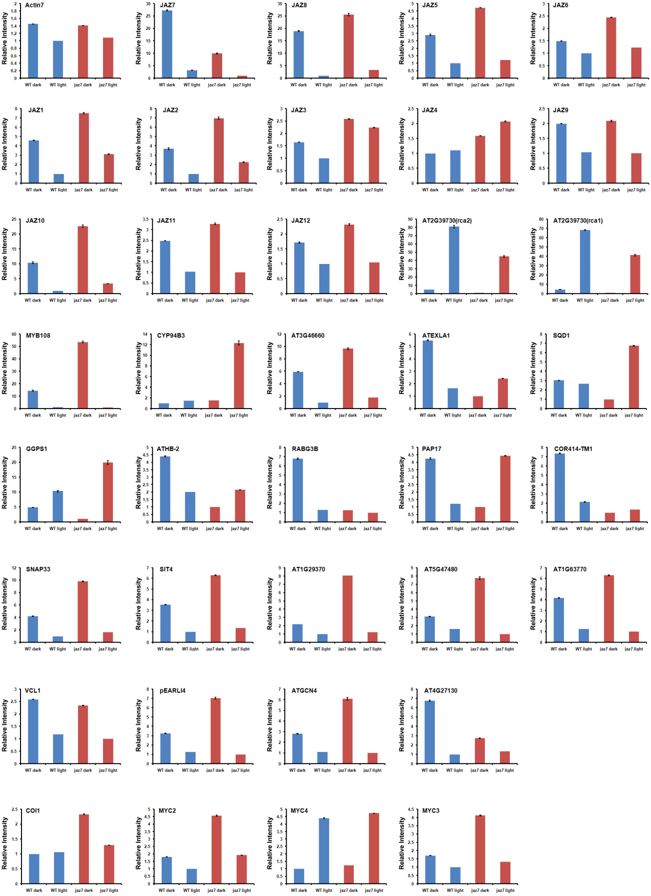
Actin7
JAZ7
JAZ8
JAZ5
JAZ6
JAZ1
JAZ2
JAZ3
JAZ4
JAZ9
JAZ10
JAZ11
JAZ12
AT2G39730(rca2)
AT2G39730(rca1)
MYB108
CYP94B3
AT3G46660
ATEXLA1
SQD1
GGPS1
ATHB-2
RABG3B
PAP17
COR414-TM1
SNAP33
SIT4
AT1G29370
AT5G47480
AT1G63770
VCL1
pEARLI4
ATGCN4
AT4G27130
COI1
MYC2
MYC4
MYC3
Relative Intensity
WT dark
WT light
jaz7 dark
jaz7 light

Supplemental Table 1. Expression pattern of JAZ family genes under dark treatment

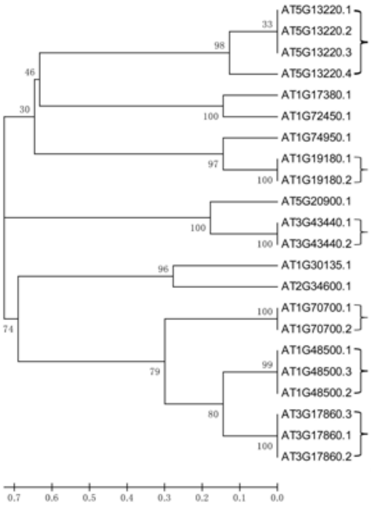

JAZ gene name (Probe Set ID)	Ratio (real time RT-PCR) Dark 3d/CK	Reference Data Mining: Ratio (Microarray[a])		Reference Data Mining: Ratio (real time RT-PCR[b])		
		Dark 1d/CK	Dark 5d/CK	Dark 2d/CK	Dark 4d/CK	Dark 6d/CK
**JAZ10 (250292_at)**	10.39 ± 0.317	3.38	7.13	4.00	4.39	3.91
**JAZ5 (261033_at)**	2.91 ± 0.052	2.31	1.95	1.48	0.80	0.87
**JAZ6 (260429_at)**	1.50 ± 0.006	1.45	1.00	0.93	0.72	0.45
**JAZ2 (262171_at)**	3.70 ± 0.112	2.65	2.64	1.29	1.18	0.95
**JAZ1 (256017_at)**	4.59 ± 0.046	3.15	8.92	1.87	1.87	1.05
**JAZ12 (246161_at)**	1.72 ± 0.019	1.50	2.02	0.72	1.00	0.68
**JAZ11 (no probe set)**	2.39 ± 0.009			0.87	1.66	0.68
**JAZ8 (256159_at)**	18.99 ± 0.138	7.85	15.75			
**JAZ7 (266901_at)**	8.40 ± 0.057	4.01	9.59	5.04	6.06	5.92
**JAZ9 (260205_at)**	1.91 ± 0.004	2.66	2.41	0.93	1.12	0.55
**JAZ4 (no probe set)**	0.91 ± 0.001			0.59	0.91	0.72
**JAZ3 (258189_at)**	1.65 ± 0.005	1.65	2.08	0.85	0.56	0.51

(a) data from Lin and Wu (in Plant J. 39:612-28)

(b) data from Parlitz S et al (in J Plant Physiol. 168:1311-9)

Supplemental Table 2. ANOVA tables for pair-wise comparisons in Figure 2F

Comparison		df	SS	MS	F	P-value
WT vs *jaz7*	Treatment	1	46855.09	46855.09	2453.463	**3.01E-15**
	Genotype	1	2590.439	2590.439	135.6426	**6.74E-08**
	Treat×Geno	1	1245.966	1245.966	65.24227	**3.41E-06**
	Residuals	12	229.1703	19.09753		
WT vs *35S::JAZ7/jaz7*	Treatment	1	42821.38	42821.38	2502.253	**2.67E-15**
	Genotype	1	95.16493	95.16493	5.56093	0.036172
	Treat×Geno	1	664.1527	664.1527	38.80954	**4.39E-05**
	Residuals	12	205.3576	17.11313		
WT vs *35S::JAZ7/WT-1*	Treatment	1	15162.44	15162.44	301.7756	**7.18E-10**
	Genotype	1	7314.503	7314.503	145.5794	**4.55E-08**
	Treat×Geno	1	3367.047	3367.047	67.01379	**2.97E-06**
	Residuals	12	602.9291	50.24409		
WT vs *35S::JAZ7/WT-2*	Treatment	1	19787.12	19787.12	1024.891	**5.45E-13**
	Genotype	1	6388.669	6388.669	330.9067	**4.21E-10**
	Treat×Geno	1	1639.88	1639.88	84.93899	**8.59E-07**
	Residuals	12	231.6787	19.30656		
jaz7 vs *35S::JAZ7/jaz7*	Treatment	1	58676.11	58676.11	3895.683	**1.89E-16**
	Genotype	1	3678.617	3678.617	244.2344	**2.43E-09**
	Treat×Geno	1	90.76534	90.76534	6.026183	0.030324
	Residuals	12	180.7419	15.06183		
jaz7 vs *35S::JAZ7/WT-1*	Treatment	1	25101.37	25101.37	520.8532	**2.97E-11**
	Genotype	1	18610.75	18610.75	386.173	**1.71E-10**
	Treat×Geno	1	8709.463	8709.463	180.7213	**1.35E-08**
	Residuals	12	578.3134	48.19278		
jaz7 vs *35S::JAZ7/WT-2*	Treatment	1	30963.66	30963.66	1794.448	**1.94E-14**
	Genotype	1	17115.31	17115.31	991.8898	**6.61E-13**
	Treat×Geno	1	5744.681	5744.681	332.9235	**4.06E-10**
	Residuals	12	207.0631	17.25526		
35S::JAZ7/jaz7 vs *35S::JAZ7/WT-1*	Treatment	1	22173.3	22173.3	479.8545	**4.81E-11**
	Genotype	1	5741.035	5741.035	124.2423	**1.1E-07**
	Treat×Geno	1	7022.008	7022.008	151.9639	**3.58E-08**
	Residuals	12	554.5006	46.20839		
35S::JAZ7/jaz7 vs *35S::JAZ7/WT-2*	Treatment	1	27701.56	27701.56	1814.014	**1.82E-14**
	Genotype	1	4924.376	4924.376	322.4688	**4.89E-10**
	Treat×Geno	1	4391.261	4391.261	287.5582	**9.49E-10**
	Residuals	12	183.2503	15.27086		
35S::JAZ7/WT-1 vs *35S::JAZ7/WT-2*	Treatment	1	6829.441	6829.441	141.0989	**5.42E-08**
	Genotype	1	31.31206	31.31206	0.646919	0.436861
	Treat×Geno	1	307.33	307.33	6.349556	0.026917
	Residuals	12	580.8218	48.40182		

df: degree of freedom; SS: stdev square; MS: mean square

Supplemental Table 4. Expression pattern of *DIN* genes for WT and *jaz7* under normal and dark treatments

Gene Name	AGI	Probe Set ID	WT dark /WT light		*jaz7* dark /*jaz7* light		WT dark /*jaz7* dark		WT light /jaz7 light	
			p-value	FoldChange	p-value	FoldChange	p-value	FoldChange	p-value	FoldChange
DIN1	AT4G35770	253161_at	**1.84E-03**	**4.62**	**5.28E-04**	**7.75**	8.18E-02	-1.25	6.31E-01	1.34
DIN2	AT3G60140	251428_at	**1.72E-05**	**57.25**	**2.94E-06**	**189.60**	1.85E-04	-1.54	8.29E-01	2.15
DIN3	AT3G06850	258527_at	**1.40E-04**	**10.52**	**2.64E-05**	**23.97**	2.26E-03	-1.44	6.10E-01	1.58
DIN4	AT3G13450	256965_at	**6.23E-04**	**15.44**	**3.76E-04**	**19.23**	2.68E-01	-1.12	9.51E-01	1.11
DIN6	AT3G47340	252415_at	**9.30E-04**	**4.02**	**1.45E-04**	**8.77**	1.29E-02	-1.37	3.37E-01	1.60
DIN9	AT1G67070	255881_at	**1.61E-04**	**18.25**	**3.74E-06**	**45.59**	**2.63E-05**	**-2.49**	9.99E-01	1.00
DIN10	AT5G20250	246114_at	**6.18E-04**	**13.75**	**1.25E-04**	**35.20**	1.02E-02	-1.44	7.54E-01	1.78
DIN11	AT3G49620	252265_at	**2.43E-02**	**31.63**	**1.10E-03**	**58.87**	**7.99E-03**	**-2.35**	9.77E-01	-1.26

Supplemental Table 5. List of primers used for real-time RT-PCR

Locus ID	Name	Forward	Reverse
	**18S rRNA**	CGGCTACCACATCCAAGGAA	TGTCACTACCTCCCCGTGTCA
AT5G09810	**Actin7**	CCAGGAATTGCTGACCGTAT	GGTGCAACCACCTTGATCTT
At2g34600	**JAZ7**	ATCCCAAACAATTCGACTCG	GGAAGTTGCTTGAATCCGAA
AT1G30135	**JAZ8**	ACCAAGTCATCCTCAAACGG	TGCTTTTGGATTTGGAAGCT
At1g17380	**JAZ5**	TTCCCTCCATCGATTCTTTG	GCTTGGGAGGATAACGATGA
AT1G72450	**JAZ6**	AAGACAGGGCTGTGGCTAGA	GGCCTGACTTTATCGATGGA
At1g19180	**JAZ1**	GAGCTTCACTTCACCGGTTC	CTTTGGCTGGATCGCATAAT
AT1G74950	**JAZ2**	CCGATTGCAAGAAGAGCTTC	GAAGACGCTTCAGCTGAACC
At3g17860	**JAZ3**	AGGCAAAGGCGATAATGTTG	CGACAGAGGCACGAGTATGA
At1g48500	**JAZ4**	TTCCCCCTCAGTTGACAATC	CAACATGATAGCTTGGGCCT
At1g70700	**JAZ9**	AGCTCTGCCACTCACACTGA	TGTTACGAGGAGCTGCATTG
AT5G13220	**JAZ10**	TCGGTAATTCTTCCGACCAC	GCTTCTCGAGAAAACGTTGC
At3g43440	**JAZ11**	GCCTTCCGTTGTTGTACGAT	AAAGAGGAGGTGCGAGATGA
At5g20900	**JAZ12**	GCCATTTCCTATTCAGCCAA	AGGAATCGTTGAAGCGAATG
AT2G39730	**RCA2**	GTTGATCAGTTCCCTGGTCAA	GCTCCTTTTCCTCTGTTTTACC
AT2G39730	**RCA1**	GTTGATCAGTTCCCTGGTCAA	GCCACAGGATCAGTACACCC
AT3G06490	**MYB108**	AGAGGATTCAGTCAGCCTCG	GGTTGTGATGCAAGATGACG
AT3G48520	**CYP94B3**	TTCTGAACCGGGGAGTACAC	TTTCCCTACACAAACCCTCG
AT3G46660	**AT3G46660**	ATAATGGAAGTCGCGTCAGG	TCTATCCACTCGGAACCAGG
AT3G45970	**ATEXLA1**	CAAAGTACCCACCGGAGCTA	CTTCCCAATTGGATGGAAGA
AT4G33030	**SQD1**	AACCGACTGGATTACGATGC	TAAGTGGGTGACCAACAGCA
AT4G36810	**GGPS1**	TCTTTCGCTTTCGAGCATTT	TTTAGCCAATTCTCCAACGG
AT4G16780	**ATHB-2**	CATGAGCCCACCCACTACTT	ACGTAGCAGCCTGAGGTTGT
AT1G22740	**RABG3B**	CGATGACTCCTTCTTGTGCA	CAACCTCCTCTTTGCTCAGG
AT3G17790	**PAP17**	AACCCAGTGACCATCAATCC	ATCTCTGCGTCCGAGTGAGT
AT1G29395	**COR414-TM1**	GGTATCAACGAGGGTGGAGA	CATCAATGCAAGGCAAGAAA
AT5G61210	**SNAP33**	GAACATGGCTGTTGACATGG	CGTTGGTTTGATTGTTGCAC
AT1G07990	**SIT4**	TCTGATATGCAAGCGAGTGG	GCCATTGGTATGTCGAGGTT
AT1G29370	**AT1G29370**	TGTCTCTCCCGGTAATTTGC	TTGGTCGAGTTTCCAAATCC
AT5G47480	**AT5G47480**	AGTAGTAGGCTCTGCAGGCG	TCTCTGCATTGCAATTGGAG
AT1G63770	**AT1G63770**	TTTTGGATCACCCAGCTTTC	CCTTGGCATGGAAATTCACT
AT2G38020	**VCL1**	CCGTGCTGCAATAAAAGTGA	GCTGCCCAGTCTTTGATTGT
AT2G20960	**pEARLI4**	GAGATGGCTGCGAAGTTAGG	AGAAGGGGTTTCCCTTGAAA
AT3G54540	**ATGCN4**	TTGGTGAGTCACGACTCGAG	ACTCTTCAAATGTGCCTGGG
AT4G27130	**AT4G27130**	AAAGAATTTTGCTGCAACGG	GACGTTCTTCCTCTGATCGC
AT2G39940	**COI1**	CTGAGACAAGGCGGCTTAAC	CCTACGTAACCCAGCAGCAT
AT1G32640	**MYC2**	AAGCTTCCGTCGTGAAAGAA	CTTCTCTACCGTTTGCTGGC
AT4G17880	**MYC4**	ATTGGTTGGGATGCAATGAT	TCGCATGATTCACTTCCAAA
AT5G46760	**MYC3**	ATACGTGTACAATGCGGCAA	TCACAACGGATAAACTCGCA
